# Lack of efficacy of imiquimod in patients with basal cell carcinoma previously treated with rituximab for B cell lymphoma: two case reports

**DOI:** 10.1186/s13256-016-0834-6

**Published:** 2016-03-11

**Authors:** Elena Campione, Monia Di Prete, Ilaria Del Principe, Laura Diluvio, Luigi Citarella, Augusto Orlandi, Sergio Chimenti, Luca Bianchi

**Affiliations:** 1 Department of Dermatology, University of Rome Tor Vergata, Viale Oxford 81, 00133 Rome, Italy; 2 Department of Hematology, University of Rome Tor Vergata, Viale Oxford 81, 00133 Rome, Italy; 3 Royal London Hospital, Whitechapel Road, London, E1 1BB UK; 4 Department of Pathology, University of Rome Tor Vergata, Viale Oxford 81, 00133 Rome, Italy

**Keywords:** Skin cancer, Immunomodulant therapy, Anti-CD20 monoclonal antibodies

## Abstract

**Background:**

Non-Hodgkin lymphomas are a heterogeneous group, which involve either B or T lymphocytes. The most used treatment is a chemotherapy regimen, which includes cyclophosphamide, hydroxydaunorubicin, oncovin and prednisone combined with rituximab - a monoclonal antibody specific for CD20 - an antigen expressed on B lymphocyte membrane. Nonmelanoma skin cancers are the most common forms in patients who have lymphomas.

**Case presentation:**

We reported the cases of two Caucasian men affected by non-Hodgkin disease, treated with chemotherapy and rituximab. After treatment, they both presented superficial basal cell carcinoma and we prescribed imiquimod 5 % cream. Unfortunately, the drug was not effective in either patient and the tumors were excised.

**Conclusions:**

We speculated about the effect of rituximab on B lymphocytes, on a particular population of T cells and on antigen-presenting dendritic cells that may have determined a lower expression of some surface antigens involved in antigen presentation. These cells are the specific targets of imiquimod to promote skin cancer cells apoptosis. A lack of action by imiquimod on skin cancer after treatment with rituximab is likely due to its transitory inhibitory effects on lymphocytes and Langherans cells. Further studies could be useful to understand the mechanism behind the lack of response.

## Background

Non-Hodgkin lymphomas (NHLs) are a heterogeneous group, which involve either B or T lymphocytes. Their causes may vary and include infectious agents, chemicals, autoimmune and genetic diseases. Clinical presentation consists in fever, night sweats, weight loss, asthenia, pruritus and superficial nonpainful lymphadenopathy. The most used treatment is a chemotherapy regimen, which includes cyclophosphamide, hydroxydaunorubicin, oncovin and prednisone (CHOP). This is usually combined with rituximab (RTX), a monoclonal antibody specific for CD20, an antigen expressed on B lymphocyte membrane [1]. Nonmelanoma skin cancers (NMSCs), including basal cell carcinoma (BCC) and squamous cell carcinoma (SCC), are common forms of malignancy in the general population, and in immunosuppressed people in particular, especially in those with a lymphoma [2]. They are more than 90 % of all skin cancers and are mostly localized in the head-neck region [3]. NHL patients are two times more predisposed to develop second primary malignancies, with men at greater risk compared to women [4]. NMSCs developed in these patients were found to be more aggressive and at risk of recurrence after Mohs microsurgery with respect to the general population, due to the impairment of the immune system during the hematologic disease and chemotherapy [2]. Mohs microsurgery, or micrographic surgery, was developed in the 1930s by F.E. Mohs and consists in microscopic examination of the tumor following its serial excision [5]. At present, patients prefer a drug treatment for BCC like imiquimod as this ensures a wider action on the field of cancerization through to the activation of antigen-presenting cells (APCs) and stimulation of T helper 1 (Th_1_) antitumoral cellular immune response [6]. In this regard, imiquimod is the most used and effective drug, especially on superficial BCC, with a successful treatment in nearly 88 % of cases [7, 8]. Imiquimod is an immunoresponse enhancer that works by activating Toll-like receptor 7 (TLR-7). The medicine is licensed in a cream form that patients use for the treatment of external genital warts, superficial BCCs, and actinic keratosis in adults [6, 9, 10]. Our experience shows a comparable percentage of success of the two, surgical and topical approaches (90–95 % vs. 85–90 %, respectively). Here we describe the cases of two unrelated men affected by NHL and treated with CHOP combined with RTX. About 6 months after RTX suspension, they presented superficial BCC of the skin.

## Case presentation

We present the cases of two Caucasian men in remission for NHL, treated with CHOP for 6 cycles (for about 4 months). They both underwent autologous stem cell transplantation after a carmustine, etoposide, cytarabine, and melphalan/cyclophosphamide (BEAM/BEAC) conditioning regimen. One of these patients was treated with RTX at the same time as CHOP, while both used a maintenance therapy for about 1 year with RTX after transplantation. They were referred to our department in their remission period and had not been using any immunochemotherapy for about 6 months. Case 1 patient is a 62-year-old man with a superficial BCC of the face (11 mm in diameter), while the case 2 patient, 47 years old, had two superficial BCCs on his back (8 and 13 mm in diameter). We prescribed imiquimod 5 % cream since both patients wanted to avoid surgical procedures. We decided to start the treatment five times a week for 8 weeks. At the end of this period, both patients did not show any response to medication. At follow-up, during the treatment, our patients did not show any typical inflammatory response to the drug. Consequently, the tumors had to be surgically removed. The histopathological examination confirmed BCC in both patients.

## Discussion

Hematologic patients are at greater risk of developing second primary malignancies, due to their immune impairment, caused by the immunosuppressive factors they produce, but also to the lymphoma treatment. According to some studies, a history of chemotherapy is a risk factor for the development of BCC [2]. The gold standard treatment is surgical excision, but in older people, patients with multiple lesions and transplanted patients, the medical approach is to be preferred. The most used topical therapies are imiquimod and 5-fluorouracil. Most cases are treated with imiquimod rather than surgically, as it has been shown that the drug acts also on the cancerization field, as demonstrated by the reduced risk of subsequent BCC in treated patients [11]. Imiquimod is an immune response modifier, which acts as a TLR-7 agonist. TLRs usually recognize pathogens and activate the releasing of inflammatory cytokines. In fact, TLR-7 triggers the transcription factor NF-kappaB, promoting the production of proinflammatory cytokines and other inflammatory mediators. This function leads to the activation of APCs and stimulation of an important Th_1_ antitumoral cellular immune response. Moreover, at higher concentrations, imiquimod causes a proapoptotic activity against tumor cells. This involves caspases activation and apparently depends on Bcl-2 proteins. This family of antiapoptotic proteins controls mitochondrial permeability. Imiquimod induces a reduction of Bcl-2 protein expression and, consecutively, an increase of the apoptotic index of the BCC cells [6]. Finally, it has been recently demonstrated that imiquimod acts directly on BCC by inhibiting Hedgehog signaling, given that this skin cancer is Hedgehog-driven [12]. The combination of all these activities explains the antitumoral action of imiquimod. In our two patients, previously treated with R-CHOP, we observed a reduced efficacy of imiquimod. This drug is used due to its effect on T cell activation, on increasing natural killer (NK) cells and on APC functions as well as against other tumors. Its efficacy has been demonstrated on cutaneous lymphomas (mycosis fungoides) [13]. Therefore, we speculate a likely effect of previous treatment with RTX on T cells. RTX acts specifically on CD20, expressed on B lymphocyte membrane with a transient effect on a particular population of T cells. This subpopulation expresses CD20 surface antigen, as well as CD3. They represent a pool of constitutive activated T cells, which produce and release a high quantity of proinflammatory cytokines [14]. Moreover, we also studied and determined the effect of RTX on the most important APCs, the dendritic cells (DCs). Apart from morphology change, there was a lower expression of some surface antigens (HLA-DR, CD80, CD83, CD86, IL-12p70) involved in antigen presentation, stimulating proliferation and inhibiting T cells apoptosis. Transforming growth factor beta 1 (TGF-beta1) increased, thus causing the inhibition of T cell action, preventing proliferation of activated T lymphocytes/activation of quiescent helper or cytotoxic T cells and the secretion of proinflammatory cytokines [especially interferon-gamma (IFN-gamma) and interleukin-2 (IL-2)]. All these mechanisms reflect a reduced ability to activate T cells by DCs [15]. We also saw a transient, dose-dependent T cell inactivation after RTX administration. Their responsiveness to DCs reduced remarkably during treatment, as shown by the higher risk of T cell-dependent infectious diseases that then reduces a few months after the end of the therapy [16]. We suppose that RTX is the cause of the lack of response to imiquimod in these patients, because CHOP has been demonstrated to cause reversible peripheral blood cytopenia. On the other hand, RTX reduces the function, as well the count of peripheral blood and DCs. RTX most likely decreases the host immune response making patients anergic toward topical immune stimulation by imiquimod. Besides B cell depletion, that could reflect the transient reduced activation of T cells, RTX acts on T cells in different ways. Despite its direct functioning on CD20+ T cells, this is only transient. The most likely mechanism of action of RTX could be due to its action on DCs. In fact, patients with an important reduction of T cell activation and functioning could develop typical T cell-dependent infectious diseases.

## Conclusions

Our clinical observation is useful for patients who undergo specific targeted therapies such as RTX to consider other therapeutic strategies in the management of NMSC different from imiquimod.

Further studies are needed to determine the real mechanism behind the lack of response to topical imiquimod in patients previously treated with this immunotherapy protocol.

## Consent

Written informed consent was obtained from the patients for publication of this case series and any accompanying images. A copy of the written consent is available for review by the Editor-in-Chief of this journal.

## Abbreviations

APC: Antigen-presenting cell
BEAM/BEAC: Carmustine, etoposide, cytarabine, melphalan/cyclophosphamide
BCC: Basal cell carcinoma
CHOP: Cyclophosphamide, hydroxydaunorubicin, oncovin and prednisone
DC: Dendritic cell
IL-: Interleukin-
INF-gamma: Interferon-gamma
NHL: Non-Hodgkin lymphoma
NK: Natural killer
NMSC: Nonmelanoma skin cancer
RTX: Rituximab
SCC: Squamous cell carcinoma
TGF-beta1: Transforming growth factor beta 1
Th_1_: T helper 1
TLR-7: Toll-like receptor 7
